# Childcare sharing and family happiness: analyzing parental and child well-being in the actor-partner interdependence model

**DOI:** 10.3389/fpubh.2024.1361998

**Published:** 2024-04-18

**Authors:** Young-Eun Lee

**Affiliations:** Department of Early Childhood Education, Gachon University, Seongnam, Republic of Korea

**Keywords:** childcare sharing, family happiness, parental well-being, child well-being, actor-partner interdependence model, korean families, gender norms, family dynamics

## Abstract

**Introduction:**

The exploration of the relationship between parental and child happiness, particularly in the context of shared childcare responsibilities, has not been examined in Korean families.

**Methods:**

Using a two-wave longitudinal design and data from 1,403 families from the Panel Study on Korean Children, this study employed the actor-partner interdependence model to examine the dynamics of childcare sharing between mothers and fathers in South Korea.

**Results:**

Mothers’ childcare sharing was found to have no significant impact on their own and their partner’s happiness, reflecting traditional gender norms that undervalue mothers’ contributions. In contrast, fathers’ childcare sharing had a positive impact on their own and their spouse’s happiness, suggesting a growing recognition of fathers’ involvement. Furthermore, fathers’ active participation in childcare was found to promote their children’s happiness through their own happiness.

**Discussion:**

This study reflects the complexity of evolving family roles and the covert persistence of traditional gender roles in modern Korean parenting. It suggests the importance of work and family policies that support changes in family dynamics by providing a more nuanced understanding of how changing family roles and responsibilities can enhance overall family well-being.

## 1 Introduction

In modern society, parental childcare is more than just the provision of basic necessities. It involves creating an environment that prioritizes children’s physical, emotional, and intellectual needs while protecting them from potential harm (1, 2). In this context, parents often seek professional advice and devote considerable time to caring for their children, emphasizing both the quantity and quality of time for sensitive and warm interactions (3, 4). Child-centered, time-intensive care provided by parents in modern societies, including careful planning and engagement in parenting activities, is positively associated with the healthy development and well-being of children. However, empirical research suggests that the relationship between time-intensive care practices and child outcomes is variable and influenced by several factors, including parents’ economic status and cultural background (4, 5). This approach focuses on dedicated time and attention and differs from intensive care, which involves a higher level of care that is sometimes required for children with special health or developmental needs. The impact of time-intensive care on the well-being of parents and children remains underexplored in current research, particularly in the context of everyday parenting.

Globally, especially in Eastern European and non-Western countries, a significant number of people view parenthood as a source of happiness and fulfillment, but longitudinal studies have shown that parenthood does not guarantee happiness (6). Studies show childcare as one of the roles of parenthood that can reduce marital satisfaction and lead to high levels of stress, anxiety, and depression (7, 8). Vanassche et al. (9) found that parenthood did not affect the happiness of either men or women. The authors suggested that negative factors associated with caregiving offset the happiness gained from parenthood; however, in societies with a high perception of parenthood, caregiving was only found to affect the happiness of men. The researchers interpreted this as being due to the disproportionate caring responsibilities of mothers compared to fathers; no matter how highly a society values parents, the benefits mothers receive do not outweigh their sacrifices. According to McDonnell et al. (10), mothers and fathers do not differ in their reports of the importance of childcare activities; however, there is a significant difference in emotional rewards, particularly in terms of happiness. Mothers often experience less happiness, more stress, and more fatigue than fathers, even though both genders rate childcare as equally important. Other research has also shown that mothers who participate in caregiving experience fewer positive emotions, such as happiness, than do fathers (11–13). It is important to note, however, that these studies were not a direct examination of childcare sharing and were conducted in Western countries. To address this, the current study specifically aims to explore the relationship between shared childcare responsibilities among parents and their happiness, and how this in turn influences children’s happiness in the South Korean context.

Although research has provided valuable insights into the dynamics of childcare and parental happiness, a notable gap remains in the context of Korean families. Despite the universal nature of the challenges and joys of childcare, cultural context plays a crucial role in shaping these experiences. In South Korea, unique social, economic, and cultural factors influence childcare sharing and perceptions of happiness in the context of childcare, and the burden of childcare is increasing due to nuclear family and the rise of dual-income families (14–16). Time-intensive and affluent parenting norms and expectations have serious implications for low fertility (2, 4, 17). According to statistics from South Korea, 56.8% of 1,300 South Korean adults reported that they are unwilling or hesitant to have children because of the high burden of care costs (18). In 2022, South Korea’s total fertility rate was 0.78, the lowest fertility rate among OECD countries in the last decade (19). Furthermore, in South Korea, the significance of this issue has been heightened by the transition to a dual-income norm, which has not fundamentally altered the gender-specific expectations rooted in Confucian culture (20). Additionally, Confucian-based gender roles, particularly the demands placed on working mothers, have intensified (21–24). Thus the focus of this study is on the dynamic interplay between childcare sharing and parental happiness within Korean families, and how these factors contribute to the overall well-being of family members. Based on this focus, the study explores two main research questions. The first question investigates whether the sharing of childcare responsibilities by Korean mothers influences the happiness of either parent and their children. The second question examines the effect of Korean fathers’ involvement in childcare on the happiness of both parents and their children.

Gender role theory provides an important lens for understanding the division of parenting responsibilities and their impact on Korean parental happiness. The core of this theory is the assumption that social structures assign different roles to men and women, influencing their behavior and attitudes (25). In the context of parenting, traditional gender roles often designate mothers as the primary caregivers. This social norm is reflected in many cultures, especially in collectivist, family-centered Eastern societies such as Korea, where mothers are expected to be primarily involved in childcare (26, 27).

Role congruence theory, which is an aspect of gender role theory, suggests that individuals tend to adopt behaviors that are consistent with their prescribed social roles (25). This congruence between social expectations and individual behavior reinforces traditional gender roles in parenting. In the case of maternal parenting, mothers often conform to social expectations of being the primary caregiver, a role traditionally assigned to women, and this has been observed in modern society as well. As Coltrane and Adams (28) pointed out, while there has been some progress in fathers’ involvement in childcare, it is important to note that this has not been a fundamental change. Modern societies, both in the East and West, have created a social climate that encourages fathers’ involvement in childcare, but the changing role of fathers has not yet significantly altered mothers’ traditional childcare practices (17).

In addition to these social constructs and expectations, the internalization of gender roles plays an important role in explaining the relationship between mothers’ shared parenting and happiness because mothers are socially conditioned to accept themselves as the primary caregivers, and they may view parenting as a natural duty and a given role. In addition, because mothers tend to have high expectations of parenthood, they may feel less happy because of potential disappointment. This may stem from the societal norm that women should find joy in motherhood and their own parenting (29, 30). This may prevent mothers from enhancing their happiness through parenting. Furthermore, because this internalization of gender roles continues to influence mothers’ role expectations and satisfaction, mothers’ childcare sharing may not increase the happiness of their partner or the mother herself.

However, fathers’ childcare sharing may increase their happiness compared to situations in which mothers care for their children. Studies have shown the benefits of fathers’ involvement in parenting (31–33). When fathers shared a significant proportion of childcare, they experienced higher levels of happiness (10), satisfaction with their lives (34), and a greater sense of belonging to the family (35). They were also more effective at work (36). Fathers’ shared parenting has a positive impact not only on fathers but also on their partners (31, 34, 36, 37). Paternal parenting helps partners feel empathy and intimacy (38), improves marital relationship quality, and reduces divorce rates (37). Some scholars have interpreted fathers’ shared parenting as a violation of their partners’ maternal identity or an encroachment on mothers’ traditional spheres of power (39, 40). However, these interpretations may be influenced by societal expectations of the father’s role (41). In recent Korean society, fathers’ childcare roles are considered as important as mothers’ roles; therefore, we did not consider them as evidence for the hypotheses of this study (42).

Exploring the relationship between parental happiness and child happiness, particularly in the context of shared childcare responsibilities, is a crucial aspect of this study. The literature has demonstrated the transfer of emotions from parents to their children, with a significant focus on the transfer of stress and negative emotions (43–45). However, recent research has begun to illuminate this positive correlation, indicating that parental happiness can predict children’s happiness (46, 47).

A notable gap in the research is the limited investigation into the combined effects of parents’ happiness on the children’s emotional state. This is especially relevant considering that earlier studies have often focused on adolescent happiness using limited measures and have not adequately explored the unique dynamics of middle childhood. This stage of development is critical, as preadolescent children’s happiness is predominantly influenced by their family environment rather than by peer interactions, and the impact of parental factors is more pronounced (48–50).

Seven-year-olds, the focal age group in this study, are particularly adept at expressing and understanding a range of emotions, making them an ideal population for examining the nuances of emotional development and happiness (51). The ability of this age group to reflect on and articulate emotional experiences offers a rich perspective on the influence of parental happiness in the family setting.

The current study aimed to fill these gaps by assessing the happiness of children in middle childhood within the specific context of childcare. This approach is underscored by recognizing the importance of children’s subjective perspectives in understanding their emotional and social relationships (52, 53). By directly assessing children’s happiness, this study overcomes the limitations of previous research that has often excluded children’s voices because of concerns about the reliability and validity of such measures (54). In doing so, this study provides a more comprehensive understanding of how parental happiness, as influenced by shared childcare responsibilities, affects children’s emotional well-being. This exploration is particularly significant given its broader implications for child development and family health, especially in the context of Korean society, where family dynamics are rapidly evolving.

Building on the literature and addressing the gaps identified, particularly in the context of South Korean families, this study aimed to explore the complex relationships between childcare sharing among mothers and fathers and its impact on family happiness. Employing the actor-partner interdependence model (APIM), this study aimed to dissect the nuanced interplay of individual behaviors and their effects within the family unit. By delineating actor and partner effects, the APIM provides a comprehensive framework for understanding how personal behaviors or characteristics influence both the individual’s and their partner’s behaviors (55). The focus is on quantifying the extent of childcare sharing from the perspectives of Korean mothers and fathers and exploring its impact on their happiness.

The underlying hypotheses of this research reflect the changing gender roles in modern society. The first hypothesis is that the sharing of childcare responsibilities between mothers and fathers has no significant impact on the happiness of either mother or father, and by extension, on the happiness of their children. This hypothesis is based on the understanding that mothers’ traditional gender roles and their internalization are still influential in modern society, which could potentially limit the impact of co-parenting on the happiness of parents and children.

In contrast, the second hypothesis is that fathers’ childcare sharing has a positive impact on the happiness of both mothers and fathers and, by extension, on the happiness of their children. This hypothesis is based on the increasing number of dual-income families in modern society and the emphasis on child-centered, time-intensive care. These factors are changing the traditional role of fathers and raising expectations that fathers’ active involvement in childcare can improve the happiness of both parents as well as their children. This hypothesis emphasizes the importance of father’s childcare sharing in shaping the overall family climate including parental happiness and children’s happiness.

By examining these aspects of childcare sharing and their effects on family members’ happiness, the study aimed to provide deeper insight into the complexities of family dynamics and the pivotal role played by shared parenting responsibilities in contemporary family life.

## 2 Methods

### 2.1 Participants

Data were obtained from the Korea Children’s Panel Survey (PSKC) of the Korea Institute of Childcare Education (KICCE). The PSKC is a prospective cohort study on the growth and development of Korean children. Since 2008, the PSKC has collected annual information from samples representing approximately 2,078 children and their families in South Korea (56). This study utilized data from 2014 (Time1) and 2015 (Time2). Time1 included 1,620 parents and 1,658 children, and Time2 included 1,560 children. For 1,403 cases at Time1 and Time2, complete data without missing information on the key and confounding variables were available. The final sample consisted of 1,403 children (49% girls) and their mothers and fathers. At Time1, the children’s ages ranged from 6 to 6.5 years old (*M* = 6.25, *SD* = 0.12). At Time2, the children’s ages ranged from 7 to 7.7 years old (*M* = 7.3, *SD* = 0.13). The ethnic composition of the sample was homogeneous, as all children were Korean. At Time1, the mothers were 25–53 years old (*M* = 36.81, *SD* = 3.70), and at Time2, they were 26–54 years old (*M* = 37.90, *SD* = 3.73). The fathers were 25–56 years old at Time1 (*M* = 39.26, *SD* = 4.00) and 26–56 years old at Time2 (*M* = 40.30, *SD* = 3.96). Thirty-nine percent of the mothers had a four-year university or higher degree, 30% had a two-or three-year college degree, and 31% had a high school diploma or less. Fifty-two percent of the fathers had a four-year university degree or higher, 21% had a two-or three-year college degree, and 27% had a high-school diploma or less. Fifty-six percent of the mothers were unemployed, whereas 4% of the fathers were unemployed. The mean family income per month was $3,416 at Time1 and $3,538 at Time2 (Table 1).

**Table 1:** Participants characteristics.

Variable	Numbers	%
1. M age at Time1 (*n* = 1,400)		
20–30 years	48	3.4
31–40 years	1,147	81.9
41–50 years	201	14.4
51–60 years	4	0.3
2. M education at Time1 (*n* = 1,320)		
High school or less	409	30.9
College degree	392	29.7
University degree	519	39.3
Higher studies	1	0.1
3. M employment at Time1 (*n* = 1,385)		
Employed	617	44.5
Unemployed	768	55.5
4. F age at Time1 (*n* = 1,398)		
20–30 years	11	0.8
31–40 years	897	64.2
41–50 years	480	34.3
51–60 years	10	0.7
5. F education at Time1 (*n* = 1,398)		
High school or less	376	27
College degree	288	20.6
University degree	586	41.9
Higher studies	148	10.6
6. F employment at Time1 (*n* = 1,403)		
Employed	1,342	95.7
Unemployed	61	4.3
7. C age at Time2 (*n* = 1,403)		
84–88 months	950	68
89–92 months	453	32
8. C gender at Time2 (*n* = 1,403)		
Girl	688	49
Boy	715	51
9. Family income at Time1 (*n* = 1,399)		
<3,000,000 won ($2,251)	210	15
3,000,000-3,999,999 won ($2,251-3,001)	335	24
4,000,000-4,999,999 won ($3,001-3,752)	331	24
>5,000,000 won ($3,752)	523	37

M, Mother; F, father; C, child.

### 2.2 Measurements

#### 2.2.1 Parents’ childcare sharing

In order to measure parents’ childcare sharing as an indication of parents’ perceptions of the level of childcare sharing, this study utilized the National Institute of Child Health and Human Development Study of Early Child Care and Youth Development Phase II Data Collection Instrument, which was translated into Korean by the PSKC research team (56). The scale consists of 16 items (e.g., taking the child to the doctor, preparing breakfast for the child, supervising the child when friends come over). The items are responded to using a 5-point Likert scale where 1 = *My partner does much more* to 7 = *I do much more*. Total scores for mothers and fathers were separately calculated by averaging partners’ reversed responses on all items. Higher scores indicate greater participation in childcare as perceived by the partner. Cronbach’s alpha was 0.85 for mothers and 0.84 for fathers.

#### 2.2.2 Parents’ happiness

Parents’ happiness was measured using the Subjective Happiness Scale (SHS) (57) and a revised version of the Kansas Marital Satisfaction Scale (RKMSS). The SHS consists of four items that are responded to using a 7-point Likert scale ranging from 1 (*not at all characteristic of me*) to 7 (*very characteristic of me*). The RKMSS, which was developed by Chung (58) and revised by the PSKC research team, consists of four items that are responded to using a 4-point Likert scale ranging where 1 = *not satisfied at all* to 4 = *very satisfied*. A one-factor confirmatory factor analysis of the two scales was performed to test the presumption that they measured the same concept (i.e., parental happiness) (59). One factor for the eight items showed a good fit, with a single common factor explaining 60% of the variance, all factor loadings above 0.6 (data not shown), and low mean residual correlation coefficient. Thus, the total scores for mothers and fathers were calculated separately by summing the SHS and RKMSS z-scores. Higher scores indicate greater parental happiness.

#### 2.2.3 Children’s happiness

The assessment of children’s happiness included three psychological dimensions, each measured using a specific scale. The SHS was utilized to assess children’s subjective happiness and consisted of four items (e.g., “In general, I think myself…,” “Compared to most of my peers, I think I am…,” “Some people are able to overcome difficulties and live happily even though they have difficulties. Are you one of them?,” “Some people are not happy even though they have difficulties and nothing sad happens to them. Are you one of them?) that were answered on using a 4-point Likert scale, where 1 = *not happy at all* and 4 = *very happy*. The average of the item responses represented the child’s subjective happiness, with higher scores indicating greater subjective happiness. Cronbach’s alpha was 0.70. Children’s overall happiness was measured using the Millennium Cohort Study Child Paper Self Completion Questionnaire (60). This scale includes six items addressing different aspects of life (e.g., schoolwork, appearance, family, friends, school, and life in general) and also uses a 4-point Likert scale. The total scores for overall happiness are calculated by averaging the responses to the items, with higher scores indicating greater overall happiness. Cronbach’s alpha was 0.67. Finally, the Rosenberg Self-Esteem Scale (61) was used to assess self-esteem. The scale consists of five items that are responded to using a 4-point Likert scale where 1 = *not at all characteristic of me* and 4 = *very characteristic of me*. Cronbach’s alpha was 0.76 scales for children’s subjective and overall happiness and self-esteem were administered using the computer-assisted personal interview technique. During home visits, the interviewers engaged with the children through questions, relevant pictures, and observations. Total scores for children’s subjective and overall happiness and self-esteem were obtained by averaging responses to the items on the scales. Higher scores indicate greater happiness and higher self-esteem.

#### 2.2.4 Covariates

Family income and level of peer conflict were included as covariates. Most studies on paternal involvement in childcare are based on small and non-representative psychological data and have observed a small number of families during infancy (38). However, this study included two control variables because it was based on large-scale panel data. Family income was included as a control variable based on studies showing the association between paternal involvement in childcare and family income and the association between adults’ happiness and income (62, 63). Peer conflict was included as a control variable because children’s social relations contribute to their self-esteem and well-being (64). Additionally, considering the high enrollment rate of 99.8% among first grade students in South Korean elementary schools, the level of peer conflict is deemed a particularly important covariate in measuring happiness, as it significantly affects the daily social interactions and overall well-being of these young students (65).

### 2.3 Analytic strategy

Descriptive statistics were calculated using IBM SPSS 26.0 to determine the demographic characteristics of the participants and perform correlation analyses between parents’ childcare sharing, parents’ subjective happiness, marital satisfaction, and children’s subjective happiness, overall happiness, and self-esteem. Skewness and kurtosis were calculated to test the normality of the distribution of each variable. Confirmatory factor analysis was conducted to test the validity, reliability, and dimensionality of the measurements. Structural equation modeling was performed to examine the relationships among parents’ childcare sharing and happiness using Mplus 8.3 (66). The maximum likelihood robust estimator was used to account for the non-normality and non-independence of the data, and the bootstrap method (1,000 bootstrap samples) was employed to test the significance of the mediation and indirect effects. The fit of the measurement and path models were evaluated according to several fit indices, and their cut-off criteria were as suggested by Hu and Bentler (67). For the comparative fit index (CFI) and Tucker-Lewis index (TLI), values above 0.95 indicate a generally good fit. Root mean square error of approximation (RMSEA) values below 0.05 indicate a good model fit, and values between 0.06 and 0.08 indicate an adequate fit. Values for the standardized root mean square residual (SRMR) below 0.08 indicate a relatively good fit.

## 3 Results

### 3.1 Descriptive statistics and correlation analysis

Table 2 presents the means, standard deviations, skewness, kurtosis, and correlation coefficients of the 10 variables that were the focus of the study. For each variable, the skewness ranged from-1.05 to 0.66, and the kurtosis ranged from −0.47 to 2.27. The distribution of each variable did not exceed the absolute values of 2 and 7, which satisfied the normal distribution assumption (68). The results of the correlation analysis showed that fathers’ childcare was positively correlated with their own and partners’ happiness, and children’s subjective happiness and self-esteem. However, mothers’ childcare was negatively correlated with their own happiness and was not significantly correlated with their partners’ or children’s happiness. Additionally, both parental happiness variables were positively correlated with children’s subjective happiness and self-esteem.

**Table 2:** Descriptive statistics and correlations between the study variables.

Variable	1	2	3	4	5	6	7	8	9	10	*M*	*SD*	S	K
1.M childcare	1										3.65	0.63	−0.81	2.27
2.F childcare	−0.37^***^	1									1.90	0.51	0.66	1.56
3.M subjective happiness	−0.06^*^	0.16^***^	1								5.25	1.04	−0.46	0.09
4.F subjective happiness	−0.05	0.16^***^	0.43^***^	1							5.33	1.01	−0.21	−0.47
5.M marital satisfaction	−0.13^***^	0.31^***^	0.53^***^	0.35^***^	1						3.23	0.64	−0.76	0.81
6.F marital satisfaction	−0.05	0.13^***^	0.38^***^	0.51^***^	0.48^***^	1					3.47	0.55	−0.83	0.66
7.C subjective happiness	−0.04	0.05^*^	0.10^***^	0.08^**^	0.10^***^	0.07^**^	1				3.21	0.54	−0.46	0.29
8.C overall happiness	0.00	0.03	0.03	0.03	0.07^**^	0.02	0.41^***^	1			3.15	0.58	−0.68	0.40
9.C self-esteem	−0.02	0.03	0.09^**^	0.09^***^	0.09^***^	0.09^***^	0.54^***^	0.41^***^	1		3.45	49	−1.05	1.53
10.C peer conflict	0.03	−0.06^*^	−0.09^**^	−0.04	−0.07^*^	−0.06^*^	−0.06^*^	−0.17^***^	−0.16^***^	1	2.32	1.32	1.00	1.56

*N* = 1,403. M, Mother; F, father; C, child; M, mean; SD, standard deviation; S, skewness; K, kurtosis.*p < 0.05, **p < 0.01, ***p < 0.001.

### 3.2 Structural equation model for the relationship between parents’ childcare sharing, happiness, and children’s happiness

The model exhibited a robust fit with the following indicators: χ^2^ = 31.781, *df* = 16, χ^2^/*df* = 1.99, *p* = 0.01, CFI = 0.99, TLI = 0.98, RMSEA = 0.027 [90% CI = 0.012, 0.040], and SRMR = 0.02. The standardized factor loadings ranged from 0.56 to 0.75. The unstandardized and standardized coefficients are presented in Table 3.

**Table 3:** Path coefficients of the structural equation model and verification of multi-mediation effect.

Parameter estimates	B (S.E.)	*β* (S.E.)
Direct effects		
Family income → M childcare	0.00 (0.00)^***^	−0.10 (0.03) ^***^
Family income → F childcare	0.00 (0.00)^***^	0.11 (0.03) ^***^
Family income → M happiness	0.00 (0.00) ^***^	0.13 (0.03)^***^
Family income → F happiness	0.00 (0.00)	0.03 (0.03)
M childcare → M happiness	0.02 (0.05)	0.01 (0.03)
M childcare → F happiness	−0.03 (0.05)	−0.02 (0.03)
M childcare → C happiness	0.00 (0.02)	−0.01 (0.04)
F childcare → M happiness	0.29 (0.06)^***^	0.15 (0.03) ^***^
F childcare → F happiness	0.58 (0.07) ^***^	0.30 (0.03) ^***^
F childcare → C happiness	0.00 (0.03)	0.00 (0.04)
M happiness → C happiness	0.02 (0.01)	0.04 (0.04)
F happiness → C happiness	0.04 (0.02)^*^	0.09 (0.04) ^*^
C peer conflict → C happiness	−0.08 (0.01) ^***^	−0.25 (0.03) ^***^
Indirect effects		
M childcare → M happiness → C happiness	0.00 (0.00)	0.00 (0.00)
M childcare → F happiness → C happiness	0.00 (0.00)	0.00 (0.00)
F childcare → M happiness → C happiness	0.01 (0.00)	0.01 (0.01)
F childcare → F happiness → C happiness	0.02 (0.01)^*^	0.03 (0.01)^*^

*N* = 1,403. M, Mother; F, father; C, child; B, unstandardized coefficient; β, standardized coefficient; S.E., standard error.*p < 0.05, ***p < 0.001.

**Figure 1:**
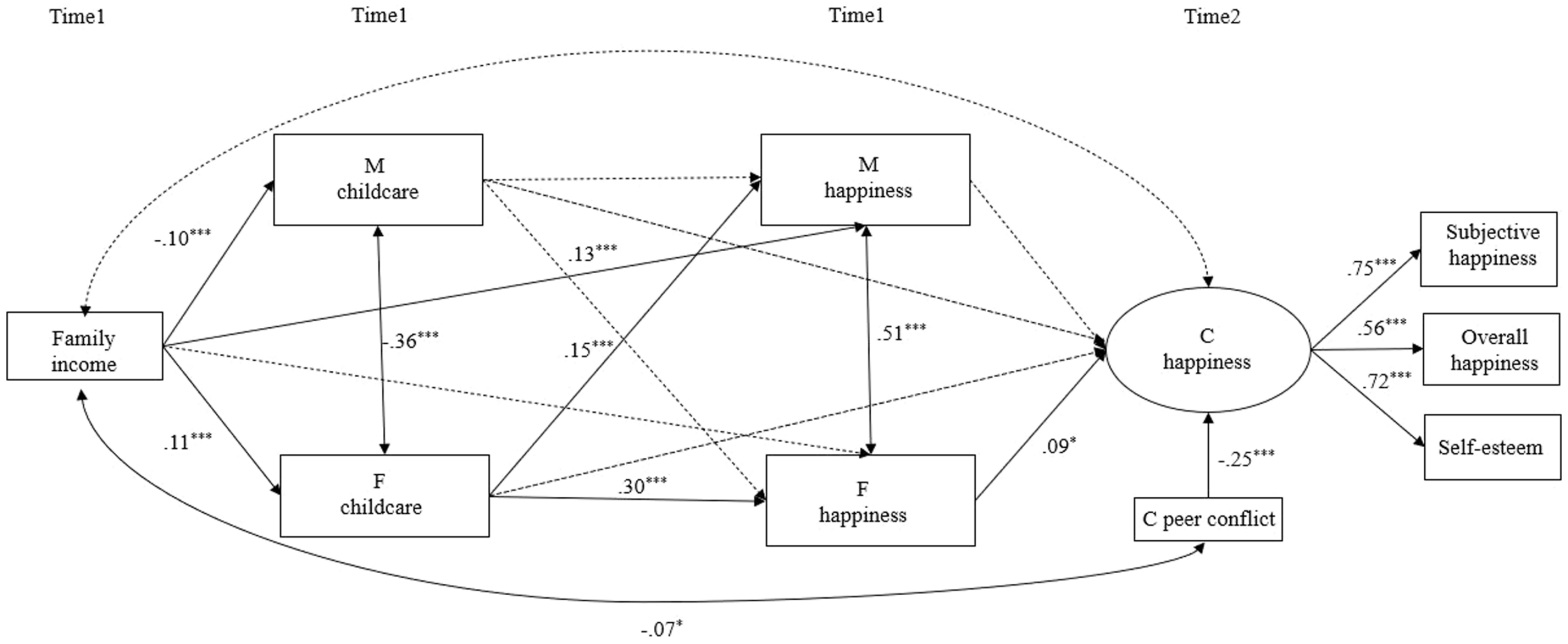
The structural equation model. Circles represent latent constructs and rectangles observable variables. All estimates are standardized coefficients. Plain arrows indicate significant paths and dotted arrows indicate non- significant paths. *p < 0.05, ***p < 0.001.

The findings from the structural equation model, depicted in Figure 1, illustrate that enhanced levels of maternal childcare do not significantly impact the happiness of mothers, fathers, or children. Conversely, an increase in paternal childcare is associated with higher happiness levels among both parents. Specifically, an elevation in fathers’ happiness has a positive effect on the happiness of children, indicating that children’s happiness is higher when fathers’ childcare is increased, mediated through the increased happiness of fathers. Bootstrapping analysis confirmed that fathers’ happiness fully mediates the relationship between fathers’ childcare and children’s happiness, with an indirect effect (*b* = 0.03, SE = 0.01, Est./SE = 2.87, *p* < 0.05).

Regarding the covariates and their relationship with the primary variables, higher family income was linked to decreased maternal childcare but increased paternal childcare. Additionally, higher family income was predictive of greater maternal happiness but had no significant predictive value for fathers’ happiness. Increased levels of peer conflict among children were associated with lower happiness levels in children.

Structural equation modeling accounted for 4% of the variance in maternal happiness, 10% of the variance in paternal happiness, 56% of the variance in children’s subjective happiness, 31% of the variance in children’s overall happiness, and 51% of the variance in children’s self-esteem, highlighting the intricate dynamics between parental childcare involvement, family income, peer conflict, and the happiness and self-esteem of children.

## 4 Discussion

Exploring the dynamics of childcare sharing between mothers and fathers in South Korea using the APIM provides important insights into family happiness. The findings on mothers’ childcare sharing and happiness indicated that mothers’ childcare involvement did not significantly affect their own, their partners’ or their children’s happiness. In other words, the first hypothesis of the study was supported. This is in line with research by Nam and Sennott (69) which highlights that the extensive role of mothers in childcare, which is deeply rooted in traditional gender role norms, is often unrecognized and undervalued in Korean families. Despite the intensive nature of modern parenting, societal expectations of mothers as primary caregivers remain entrenched, resulting in their contributions being seen as obligatory rather than extraordinary, and thus not significantly enhancing the happiness of mothers themselves, their partners, and their children.

In contrast, in line with the second research hypothesis, fathers’ sharing of childcare had a positive effect on their own and their partners’ happiness. This finding is consistent with that of Jeong and Kim (70), who reported that increased paternal involvement in childcare was associated with greater satisfaction and well-being for both parents. This reflects the changing role of fathers in South Korea, influenced by societal changes such as dual-income families and an increasing emphasis on shared parenting. Fathers’ active involvement in childcare is increasingly recognized and valued, improving the overall emotional climate of the family.

In addition, the study found that paternal involvement in childcare positively influenced children’s happiness through fathers’ happiness, which means that the second hypothesis of the study was also fully supported. This finding is consistent with that of Gerhardt et al. (71) who emphasized the direct emotional transmission from involved fathers to their children. Paternal involvement in childcare not only promotes fathers’ happiness, but also creates a positive emotional environment that benefits their children’s well-being.

Further exploration of the study’s findings in the context of the literature (72) underscores their significance. The minimal impact of mothers’ childcare on their happiness may reflect broader societal phenomena where traditional gender roles overshadow the contemporary challenges faced by mothers. This suggests a need for societal and cultural shifts that recognize and value the multifaceted role of mothers in modern parenting.

Similarly, the positive impact of paternal childcare involvement on family happiness aligns with global trends toward more egalitarian parenting roles. Studies such as McDonnell et al. (10) have highlighted how paternal engagement in childcare contributes to improved family dynamics and shared happiness, challenging traditional paternal roles and suggesting a new paradigm in family structures. Lastly, insights into how paternal happiness influences child happiness align with Park and No’s (73) study, highlighting the importance of paternal interactions with their children in fostering a nurturing and happy child environment.

However, when interpreting these results, it is important to consider the statistical power of the research model. Without including the peer conflict variable, the model explains 31–55% of child happiness, and the final model with the peer conflict variable explains 51–56% of child happiness, which affects children’s subjective happiness by 1%, but in the final model, parental happiness still has a small effect on the research model. This is expected to be due to the influence of the time of measurement of parental happiness and child happiness.

A limitation of this study includes the exclusion of certain variables from the model for simplicity. While the relationship between mothers’ childcare sharing and happiness as well as its impact on child happiness was thoroughly explored using the APIM model, factors such as mothers’ employment status, children’s gender, and birth order were not included as covariates. This decision was made to avoid overcomplicating the model given its primary focus on exploring direct relationships within the family unit. These factors are likely to have significant implications for family dynamics and happiness. Future studies should include these variables as covariates to fully understand their influence. By incorporating factors such as maternal employment, children’s gender, and birth order, future studies can provide a more nuanced understanding of how family dynamics affect parental and child happiness in different contexts.

Another limitation of this study is its focus solely on the quantitative and physical aspects of childcare, neglecting qualitative and non-physical dimensions. As highlighted by Daminger (74), women often bear a disproportionate burden of mental labor in childcare. This labor involves a complex cognitive process that encompasses anticipation, identification, decision-making, and monitoring. For example, the routine task of sending children to educational institutions requires a multifaceted approach that includes surveying the environment, identifying potential issues, making decisions, and continuous oversight. This mental load, particularly during the anticipation and monitoring stages, can lead to a significant imbalance in women’s cognitive fatigue. This study’s focus on the quantitative aspects of childcare may have overlooked these critical elements of cognitive labor that are often shouldered more by women. The numerical division of childcare responsibilities does not necessarily reflect the hidden, yet substantial mental work involved in managing and maintaining a household. Therefore, it is crucial for future research to delve into these nonphysical and qualitative aspects of childcare, especially to understand the cognitive labor involved and its impact on parental happiness. Such investigations will not only provide a more holistic view of childcare sharing, but also inform policies and practices that acknowledge and address the mental labor aspects of parenting. Finally, the present study focused primarily on the division of parental childcare sharing, with much attention to the actual division and execution of parenting tasks. Future research would benefit from measurement and research model validation on co-parenting (75, 76), including relationship dynamics between parents or caregivers, support, decision-making, and overall quality of partnership in the parenting role, to help validate broader family dynamics.

## 5 Conclusion

In conclusion, this study revealed that despite changes in family life, societal expectations of mothers as primary caregivers are still deeply rooted in traditional gender norms. This prevailing viewpoint often leads to reduced recognition of mothers’ contributions to childcare, commonly considering them as customary duties rather than notable efforts. This reflects a cultural tendency to overlook the substantial role that mothers play in child-rearing. By contrast, the role of fathers in Korean society has undergone a significant shift. Influenced by societal changes such as an increase in dual-income households and a stronger focus on shared parenting, fathers’ contributions to childcare are gaining greater acknowledgment and appreciation. This represents a notable departure from traditional paternal roles, in line with the current societal movement toward a more equitable approach to parenting.

By incorporating the perspectives of both mothers and fathers and employing the APIM, this study sheds light on the intricate interactions between gender roles, parental happiness, and child well-being. This underscores the need for balanced parenting responsibilities and adaptation to the modern Korean societal context. These findings offer a more nuanced understanding of how evolving family roles and responsibilities can enhance overall family happiness, thereby addressing the complexities and expectations of contemporary parenting.

## Data availability statement

Publicly available datasets were analyzed in this study. This data can be found at: https://kicce.re.kr/pskc/module/rawDataManage/index.do?menu_idx=56 Panel Study on Korean Children.

## Ethics statement

The studies involving humans were approved by Gachon University Institutional Review Board. The studies were conducted in accordance with the local legislation and institutional requirements. Written informed consent for participation in this study was provided by the participants’ legal guardians/next of kin.

## Author contributions

Y-EL: Conceptualization, Formal Analysis, Funding acquisition, Project administration, Resources, Supervision, Validation, Visualization, Writing – original draft, Writing – review & editing.
